# Spliceosome inhibitor induces human hematopoietic progenitor cell reprogramming toward stemness

**DOI:** 10.1186/s40164-022-00288-9

**Published:** 2022-06-10

**Authors:** Liaoliao Dong, Chuijin Wei, Shumin Xiong, Ping Yu, Ren Zhou, Lin Cheng

**Affiliations:** Shanghai Institute of Hematology, State Key Laboratory of Medical Genomics, National Research Center for Translational Medicine at Shanghai, Ruijin Hospital, Shanghai Jiao Tong University School of Medicine, Shanghai, China

**Keywords:** Cell reprogramming, Human hematopoietic stem/progenitor cells, Spliceosome, Small molecule compound, Lineage tracing

## Abstract

The application of hematopoietic stem cells (HSCs) has been restricted due to limited cell sources and conventional methods for generating these cells by cell expansion and pluripotent stem cell differentiation have not been clinically achieved. Cell reprogramming technique provides a new hope for generating desirable cells. We previously reported that mouse differentiated hematopoietic cell reprogramming could be induced by small molecule compounds to generate hematopoietic stem/progenitor-like cells, whether the human hematopoietic cells could also be reprogrammed into HSCs by chemical compounds remains elusive. Here, we demonstrated for the first time that human committed hematopoietic progenitors could be reprogrammed into multipotent progenitors by spliceosome inhibitor. Combination of single cell RNA-sequencing and genetic lineage tracing including exogenous barcodes and endogenous mitochondrial DNA mutations confirmed the reprogramming procession. Although the small chemical compound inhibiting spliceosome function only induces the differentiated hematopoietic progenitors to acquire plasticity and reprograms them into multipotent progenitors but not stem cells so far, this study still provides a proof-of-concept strategy for generating HSCs based on combining two independent steps together in future, first differentiating rare HSCs into large number of progenitors then reprogramming these progenitors into huge number of HSCs. Further dissecting the mechanism underlying spliceosome inhibitor-induced human hematopoietic cell reprogramming in future will help us comprehensively understanding not only the chemical reprogramming to generate desirable human cells for clinical translation but also hematopoiesis under physiological and pathological conditions.

**Supplementary Information:**

The online version contains supplementary material available at 10.1186/s40164-022-00288-9.

To the Editor

Hematopoietic stem cells (HSCs) hold great promise in the field of fundamental research and clinical application. However, the application of HSCs has been limited to rare cell source. Conventional strategies for generating HSCs have not been successfully translated into clinical application due to diverse limitations and side effects. Converting easily generated hematopoietic progenitor cells or mature cells into stem cells through cell reprogramming, provides us with a new way to obtain a large number of HSCs.

Recently, advances in single-cell sequencing, cell barcoding and lots of other technologies have improved our understanding of hematopoietic stem cell [1–3], while help us to discover more regulators of cell fate for hematopoietic reprogramming. Small molecule compounds-induced cell reprogramming has been a cutting-edge technique for generation of desirable cells [4]. Distinct advantages of small molecule compounds including cell permeability, clinical safety, reversibility, and scalability, endow chemical reprogramming with great potential for stem cell regeneration medicine [5]. We previously reported for the first time that a cocktail of small molecule compounds could reprogram mouse fibroblasts into hemogenic cells and reprogram differentiated mouse hematopoietic cells into hematopoietic stem/progenitor-like cells [6, 7]. However, whether the human hematopoietic cell reprogramming could be induced by chemical compounds to generate HSCs remains to be further investigated.

It has been demonstrated that large increase in the number of CD34^+^ cells can be achieved by culturing cells in serum-free media supplemented with cytokines. CD34 is a recognized surface marker which help us to isolate hematopoietic stem and progenitor cells (HSPCs). We isolated CD34^+^ cells from human umbilical cord blood and cultured them in vitro for one week, about half percent of the cells became CD34^−^. In the CD34^+^ cells, HSC percentage remained less than 1% and the other cells were multipotent progenitors (MPPs), common lymphoid progenitors (CLPs), common myeloid progenitors (CMPs), granulocyte-monocyte progenitors (GMPs), and megakaryocyte-erythroid progenitors (MEPs), according to FACS analysis of cell surface markers (Additional file 1: Figure S1A). The total cell number of these progenitors increased almost eight times compared with that of initial HSCs (Additional file 1: Figure S1B). RNA-sequencing of these HSCs and progenitors demonstrated that expression levels of many splicing factors gradually increased along with HSC differentiation into multipotent progenitors and committed progenitors (Additional file 1: Figure S1C). The gene expression pattern of these splicing factors in early stage of hematopoiesis is similar to that in embryonic stem cell development. Spliceosomal repression with inhibitor Pladienolide B (PlaB) could reprogram pluripotent stem cells into totipotent stem cells [8]. All these preliminary data drove us to hypothesize that spliceosomal repression might also reprogram hematopoietic progenitor cell back into HSCs. We treated the cultured CD34^+^ cells with PlaB and found that the subpopulation percentage of CD34^+^ cells, CD34^+^CD38^−^ cells, CD34^+^CD38^−^CD45RA^−^ cells, and CD34^+^CD38^−^CD45RA^−^CD90^−^ cells gradually increased with PlaB concentration from 1 nM, 3 nM to 10 nM (Fig. 1A, Additional file 1: S1D). Colony formation assay (CFU) also showed that the progenitors treated with PlaB could generate more colony number, especially for CFU-GEMM, than that in control (Fig. 1B). Transcriptomic profiling of the progenitors became closer to that of HSCs when PlaB concentration went higher, although the expression pattern of HSCs and the 10 nM PlaB group were still different (Fig. 1C–E). Together, it indicates that spliceosome inhibitor could block hematopoietic progenitors’ further differentiation or reprogram them back toward stemness.

**Fig. 1:**
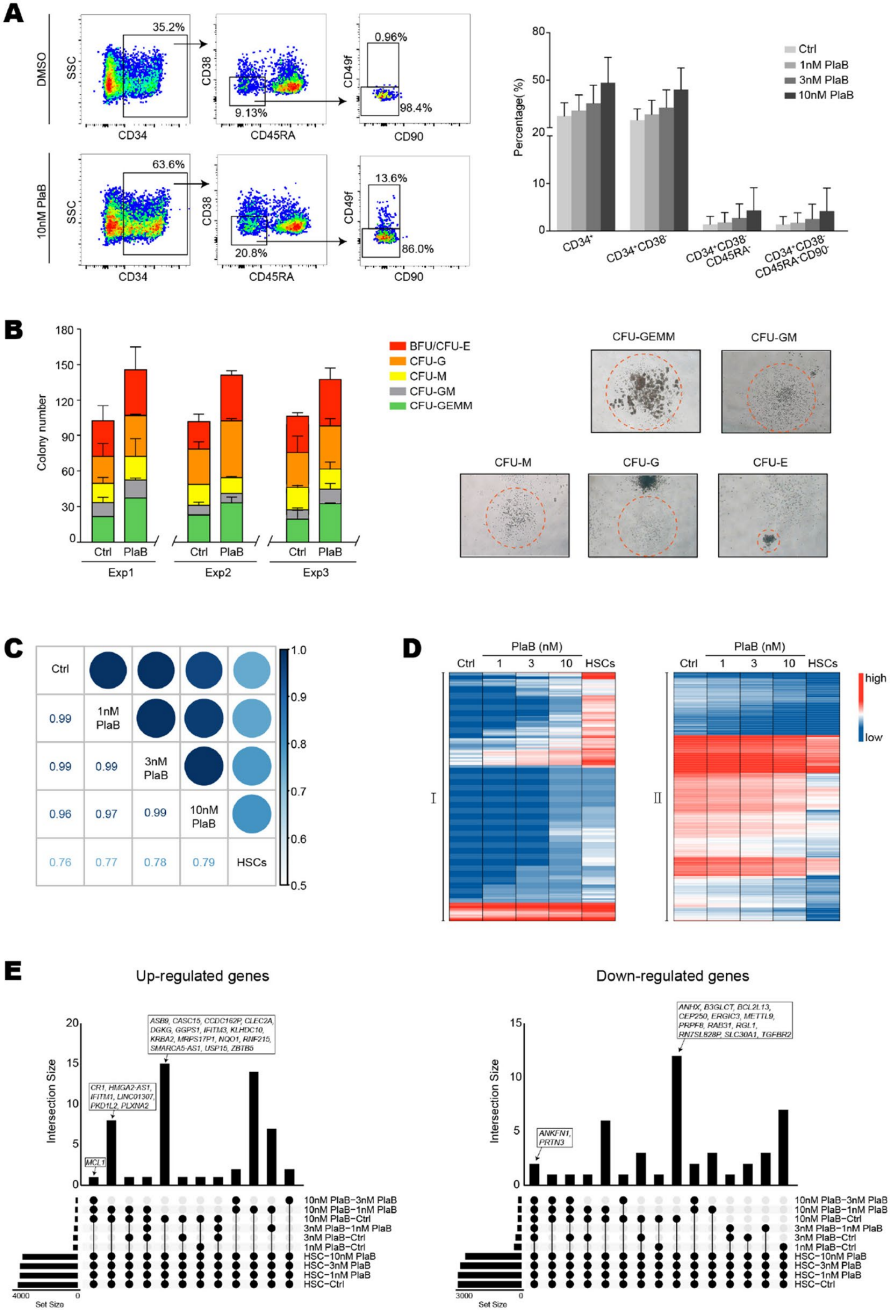
PlaB enhanced the stemness of human hematopoietic progenitors or blocked their differentiation in vitro*.* **A** FACS analysis of cells treated with PlaB for 4 days. Representative FACS plot showed the enrichment of the HSPC population in cell samples treated with 10 nM PlaB versus DMSO as control for 4 days (left). Percentage variation of HSPCs among samples treated with different PlaB concentrations were quantified (right), represented as means ± SDs from N = 3 samples. **B** Quantification of colonies from CFU assay. Cells treated with 10 nM PlaB were being with enhanced ability of colony formation compared with the control. N = 3 and data represented as means ± SDs (left). DSMO versus PlaB: 2-tailed unpaired t test with unequal variance. In terms of total CFU numbers there was no significant difference between the two group (p > 0.05). Representative colony forms were shown (right). **C** Corplot showed the sample–sample similarities among human HSCs and cells treated with different concentrations of PlaB based on bulk RNA-seq. PlaB treated cells were more closely correlated with HSCs compared with the control. **D** Heatmap of differentially expressed genes. Cluster I (left) or cluster II (right) genes have gradual up-regulation or down-regulation trends respectively in Ctrl-1 nM PlaB-3 nM PlaB-10 nM PlaB-HSCs samples based on bulk RNA-seq. Genes showing high and low-expression in the heatmap were shown in red and blue, respectively. **E** The statistics of differentially expressed genes correspond to the cluster I (left) and cluster II (right) in Fig. 1D, calculated by edgeR among the sample–sample comparisons

To distinguish the two types of possibilities, we performed single cell RNA-sequencing (scRNA-seq) combining with two different strategies of genetic lineage tracing (Additional file 1: Figures S2A–C, S3A, S4A). CellTagging technique with heritable and transcriptable tags of 8 bp random nucleotides has been utilized to trace cell fate change (Additional file 1: Figure S3A–C) [7, 9]. Besides, we further found and measured mitochondrial DNA mutations in these single cells to help verify the analyzing results from CellTags (Additional file 1: Figure S4A, B), which was developed recently for hematopoietic cell lineage tracing [10]. Accordingly, hierarchical clustering of mitochondrial genotyping profiles for the two timepoint cells was shown respectively in Additional file 1: Figure S4C. Further lineage tracing analysis was carried out with these two methods parallelly. Alignment of CellTagging and mitochondrial DNA mutations individually between the two timepoints (Fig. 2A), combining with cell type identification Additional file 1: Figure S2C), demonstrated that PlaB could not inhibit hematopoietic progenitor cell differentiation. More mature hematopoietic cells were detected at the second timepoint, including NK cells, eosinophils, basophils, mast cells, monocytes, and dendritic cells. Pseudo-time analysis showed that the bipotent or unipotent differentiation potential of these progenitors were disturbed, such as myeloid progenitor cells could differentiate into lymphoid cells and lymphoid progenitor cells could acquire myeloid differentiation potential (Fig. 2B). We found that MPPs could be induced from CLPs, GMPs, MEPs, and CMPs, and Sankey diagram showed that each type of progenitor cells acquires different reprogramming efficiency (Fig. 2C, left). Principal component analysis confirmed these cell transitions (Fig. 2C, right). These data suggested that PlaB treatment might induce the hematopoietic progenitors to acquire plasticity, which is considered as a consequence of cell reprogramming and similar phenomenon has been observed in other somatic cell reprogramming induced by diverse factors [11]. Furthermore, we found that MPPs could be induced from CLPs, GMPs, MEPs or CMPs based on calculation by partition-based graph abstraction algorithm (Fig. 2D, Additional file 1: S3D and S4D).

**Fig. 2:**
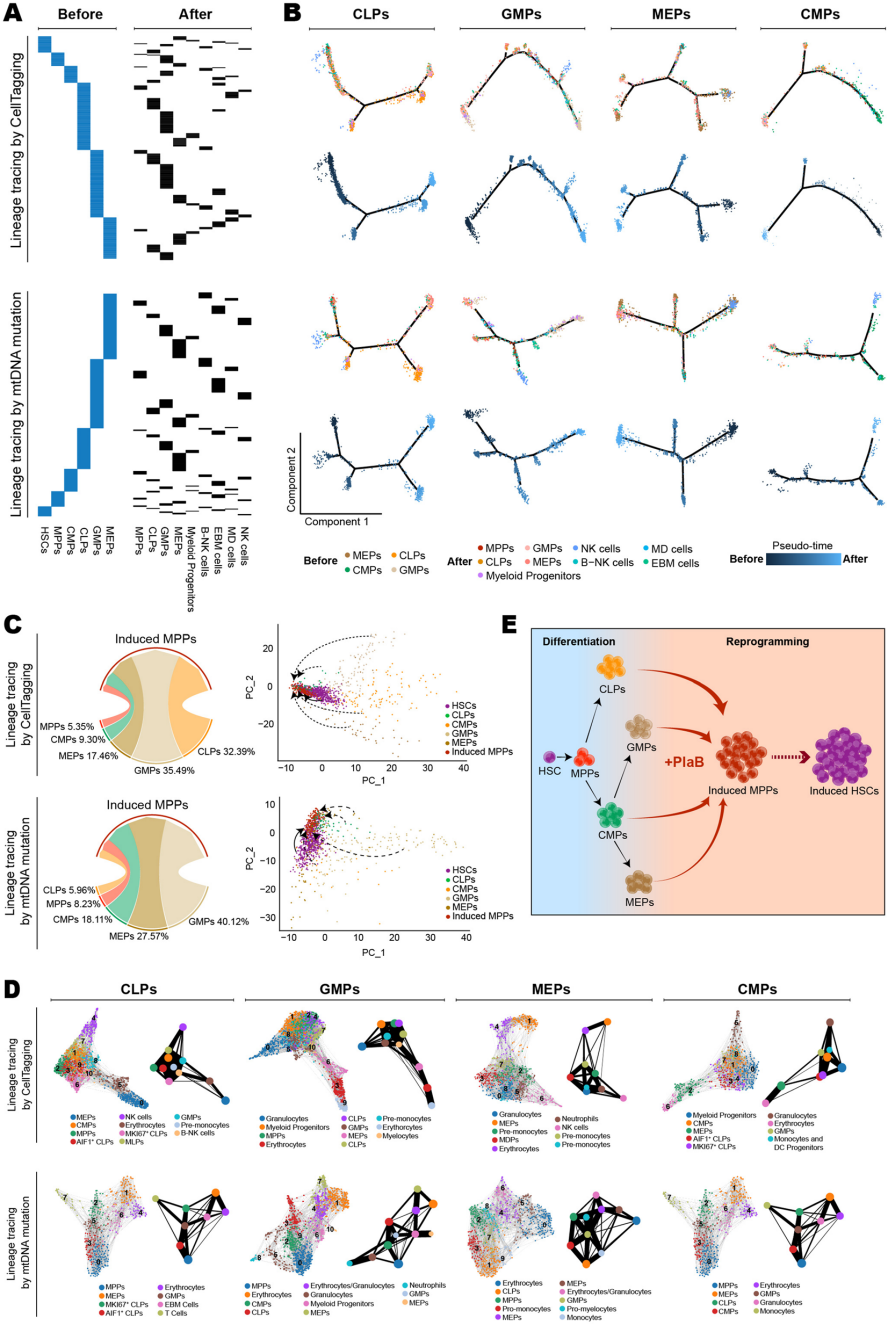
Lineage tracing by CellTagging and mitochondrial DNA mutations. **A** Correspondence between cells before and after treatment with PlaB was traced by CellTagging (up) and mitochondrial DNA mutations (down). Each horizontal line stands for a unique CellTag group (up) or a similar mitochondrial DNA mutation group (down). B-NK cells: B cells and NK cells. EBM cells: Eosinophils, Basophils and Mast cells. MD cells: monocytes and dendritic cells. **B** The visualization of major trajectories of progenitor cells and their derived cells was defined. Cells were traced by CellTagging (up) or mitochondrial DNA mutations (down). Charts were colored according to cell types or pseudo-time values. **C** Sankey diagram (left) showed induced MPPs’ origins. PCA diagram (right) showed the correlations among HSCs, CLPs, CMPs, GMPs, and MEPs before PlaB treatment and induced MPPs. **D** The visualization of clustering and PAGA trajectories of the cells derived from progenitor cells with PlaB treatment. They were traced by CellTagging (up) or mitochondrial DNA mutations (down). Charts were colored by cell types. The size of the circle represented the cell quantity and the thickness of the line represented the correlation between clusters. **E** Schematic model of the PlaB-induced human hematopoietic progenitor cell reprogramming toward stemness

In summary, it is found that spliceosome inhibitor can induce human committed hematopoietic progenitor cells to reprogram into multiple progenitors. It is the first report about human hematopoietic cell reprogramming induced by small molecule compound, which not only expands the scope of chemical reprogramming for generating desirable cell types to therapeutic application, but also helps comprehensively understand normal and neoplastic hematopoiesis [12]. Most importantly, this study suggests a very promising strategy with combining two previously independent steps together for human HSC generation (Fig. 2E).

## Supplementary Information

**Additional file 1: Figure S1.** Expression levels of splicing factors increased along with HSC expansion. **A** Representative FACS plot(left) and pie diagram(right) showed the percentage of HSPC subpopulations of human UCB HSCs after 7-day-cultured in vitro. Cell surface markers used to gate cell populations were listed. **B** Folds change of cell quantity compared with that of initial HSCs after 7-day-cultured in vitro. **C** Heatmap of splicing factors expression levels in HSCs, Multipotent Progenitors and Committed Progenitors. Many splicing factors gradually increased along with HSC differentiation. **D** Percentage variation of HSPC among samples treated with different PlaB concentrations, supplement to figure 1A. Data represented as means ± SDs from N = 3 duplicates. 2-tailed unpaired t test with unequal variance; n.s.: p > 0.05, *p < 0.05, **p < 0.01, ***p < 0.001. **Figure S2.** Basic information of sc-RNAseq. **A**, **B** Distribution of confidently mapped reads information on scRNA-seq. scRNA-seq of the cells at both timepoints before and after PlaB treatment included 11,048 and 12,173 individual cells together with 2933 and 3054 median genes and 37,771 and 29,461 mean confidently mapped reads per cell. **C** UMAP visualization based on 10× scRNA-seq before and after PlaB treatment. MD cells: Monocytes and Dendritic cells, EBM cells: Eosinophils, Basophils and Mast cells. **Figure S3.** Basic information of CellTagging and lineage tracing. **A** The CellTagging workflow: a lentiviral construct contains a heritable 8-bp random CellTag barcode in the 3’ UTR of GFP, followed by an SV40 polyadenylation signal. Transduced cells express unique CellTags, enabling tracking of clonally related cells. **B** Number of CellTags detected in scRNA-seq samples. The number of celltag inserted in each cell was from 1 to 6 and the average number was 1. **C** Number of paired and individual CellTags detected in scRNA-seq samples before and after PlaB treatment. 7888 cells before treatment and 9570 cells after that have been detected with celltags and 6290 cells between two timepoints were inserted with the same celltag. **D** Topological map of cells tracing by CellTagging, related with Figure 2D. Specific genes of each cluster were shown in PAGA layout. **Figure S4.** Basic information of mitochondrial DNA mutation and lineage tracing. **A** The principle of lineage tracing by mitochondrial DNA mutation. Each cell has multiple mitochondria, which in turn contain many copies of mtDNA that may acquire somatic mutations over time. Correlation analysis of mutation patterns enable tracking of clonally related cells. **B** Based on the scRNA-seq data, distribution of coverage of the mitochondrial genome, numbers of mutations, frequency of mutations and numbers of mutations per cell were calculated. **C** Hierarchical clustering of mitochondrial genotyping profiles (rows) for cells on two timepoints. **D** Topological map of cells tracing by mitochondrial mutation, related with Figure 2D. Specific genes of each cluster were shown in PAGA layout.

## Data Availability

The RNA sequencing data are available from the corresponding author on reasonable request.

## Abbreviations

HSCs: Hematopoietic stem cells
HSPCs: Hematopoietic stem/progenitor cells
PAGA: Partition-based graph abstraction
MPPs: Multipotent progenitors
CLPs: Common lymphoid progenitors
CMPs: Common myeloid progenitors
GMPs: Granulocyte-monocyte progenitors
MEPs: Megakaryocyte-erythroid progenitors
CFU: Colony formation assay
scRNA-seq: Single cell RNA-sequencing
