# Supplementary material for: Spliceosome inhibitor induces human hematopoietic progenitor cell reprogramming toward stemness
Source: Exp Hematol Oncol. 2022 Jun 10;11:37. doi: 10.1186/s40164-022-00288-9 (PMC9188107; doi:10.1186/s40164-022-00288-9)

**Additional file 1 data**

**Experimental Section:**

*HSCs isolation*: Umbilical cord blood samples were provided by Shandong Cord Blood Bank, China, and for all of our experiments, human HSCs were isolated from fresh umbilical cord blood that were collected within 30 hours. Briefly, mononuclear cells were isolated using Ficoll-Paque PLUS ([Cytiva](https://cdn.cytivalifesciences.com/dmm3bwsv3/AssetStream.aspx?mediaformatid=10061&destinationid=10016&assetid=12637)), then CD34^+^ cells were isolated by Miltenyi human CD34 MicroBead Kit according to the manufacturer’s instructions. All experiment was carried out according the Ethical Committee of Ruijin Hospital Affiliated to Shanghai Jiao Tong University School of Medicine.

*HSCs expansion and PlaB treatment*: Primary CD34^+^ cells were cultured in Stemspan SFEM Ⅱ (STEMCELL Technologies) supplemented with 100 ng/ml human SCF (PeproTech), 100 ng/ml human Flt3-Ligend (PeproTech), 20 ng/ml TPO (PeproTech) and 2.5% Penicillin-Streptomycin (Gibco). Every 1-2 days the cells were re-plated in fresh medium. At the day 7 of *in vitro* expansion, cells were collected and CD34^+^ cells were isolated by Miltenyi human CD34 MicroBead Kit. Sorted cells were randomized grouped and cultured in abovementioned medium with different concentrations of PlaB or an equal volume of DMSO as control for 4 days. Every 2 days the cells were re-plated in fresh medium. Changes of cell surface makers and transcriptomic profiling were detected under 1 nM, 3 nM and 10 nM PlaB treatments. CFU assay used samples with 10 nM PlaB treatment and the control.

*Flow Cytometry and Fluorescence activated Cell Sorting*: For surface maker detection, collected cells were stained with FITC Anti-Human CD34 (BD), APC-H7 Anti-Human CD45RA (BD), BV421 Anti-Human CD38 (BD), APC Anti-Human CD90 (BD), PE Anti-Human CD49f(BD), BV650 Anti-Human CD123 (BioLegend) and 7-AAD (BD) according to the manufacturer’s instructions. Multicolor analyses of cells were performed on BD LSRFortessa X-20. Results were analyzed by Flowjo software. For GPF^+^ cells isolating, collected cells were washed and resuspended in DPBS, stained with 7-AAD (BD) according to the manufacturer’s instructions, and filtered. Fluorescent cell sorting was performed on BD Aria Ⅲ SORP. Sorted cells were centrifuged to collect for follow up treatment. *Colony Formation Assay*: Collected cultured cells, resuspend evenly and counted. Transferred cells into 12-well culture plate in 1 ml MethoCult SF H4436 (STEMCELL Technologies) supplemented with 1% Penicillin-Streptomycin (Gbico) seeded at a density of 600, 800 or 1000 per well, and added IMDM (ThermoFisher Scientific) supplemented with 2% Fetal Bovine Serum (Gibco) up to 1.2 ml. Colonies were identified and counted after 10-14 days.

*Lentivirus production*: 293T cells were transfected with pSMAL-CellTag construct V1 along with packaging plasmid psPAX2 and pMD2G by jetPRIME® transfection reagent to produce lentivirus expressing the CellTags and EGFP following manufacturer’s instructions. Supernatant was collected and immediately filtered through a low-protein binding 0.45 μm filter.

*CellTagging methodology*: During the 7-day expansion *in vitro*, HSCs were infected with lentivirus expressing the CellTags and EGFP every 12 hours for 3 times at the day3 to day6. At the day 7, CD34^+^EGFP^+^ cells were isolated by fluorescent cell sorting and Miltenyi human CD34 MicroBead Kit as described above. Half of these cells were sent to scRNA-seq immediately. The rest cells were treated with 10 nM PlaB for 4 day and then the whole cells were harvested for scRNA-seq again.

*Bulk RNA-sequencing and analysis*: Cells collected after 0, 1, 3, 10 nM PlaB treatment and CD34^+^cells isolated freshly from human UCB were detected on Illumina HiSeq 2000 system individually. The FASTQ files of the cells of different treatments were aligned with the human reference(hg19) by HISAT2. The sam files, the output of HISAT2, were transferred to the bam format by samtools. Then the reads were counted by the ‘htseq-count’ function with the annotation (gencode.v38.annotation.gtf).

The correlation values of different treatments were calculated and the differential expressed genes were found by R-package edgeR. After turning the reads counts into DGEList object, the up-regulation genes and the down-regulation genes were calculated as the square-root-dispersion was set 0.1.

*Single cell RNA-sequencing and analysis*: Cells collected before and after PlaB treatment were detected on the Chromium system (10×Genomics) individually. The reads of single-cell sequencing FASTQ files were aligned to human reference dataset (GRCh38) by Cell Ranger (v3.1.0) provided by 10×Genomics to generate feature-barcode matrixes on the Shanghai Jiao Tong University HPC.

The single-cell sequence feature-barcode matrixes were analyzed by R-package Seurat (v 3.1.5) on R studio platform of SJTU HPC following the workflow on the SATIJA LAB. Each Seurat object obtained about 11,000~12,000 single cells. The top 2000 highly variable genes were chosen and the Seurat object data was scaled. The filtered, normalized and scaled Seurat objects were calculated to perform principal component analysis (PCA). Dimension of reduction to compute nearest neighbor graph used the first 20 principle components and resolution to topological divide cells into different clusters was adjusted to 1. To illustrate the definite trend, PC 1 and PC 2 were chosen to show the process of reprogramming. The Uniform Manifold Approximation and Projection for Dimension Reduction (UMAP) visualization was performed to run non-linear dimensional reduction of the Seurat object. Differential expressed genes (DEGs) were calculated by cluster and played a significant role in the following analysis such as cell type identification.

The 8-bp random CellTags inserted in cells and the mitochondria mutations were calculated on the SJTU HPC. The CellTags were searched in the bam files of the output of CellRanger. Then according to the cell barcodes, the relationship of the cells in two timepoints had been built. The mitochondria mutations were calculated with the reference built before by Pysam, which is a python module for reading, manipulating and writing genomic data sets. After getting the mutations of every cell, the relationship of the cells in two timepoints had been built. There were 6290 pairs in CellTags tracing and 5835 pairs in mitochondria mutations tracing respectively.

Scanpy, an analysis toolkit for single-cell sequence in python, was used for reconstructing the differentiation trajectory. The preprocessed feature-barcode matrix of the cells induced from the progenitors (tracing according to the CellTags and the mitochondria mutations respectively) was transferred into loom format to reconstruct the trajectory in Scanpy. The mean value of highly variable genes varied from 0.0125 to 3. PCA was computed based on the chosen variable genes. After preprocessing and PCA, the cells were clustered by Leiden algorithm and the neighborhood graph was embedded in 2 dimensions using Factor Analysis (FA). After the relationship of the cluster was constructed by partition-based graph abstraction (PAGA) algorithm, the FA visualization and the PAGA visualization were recomputed to embed for the reprogram trajectory. Resolution of PAGA to topological divide cells into different clusters was adjusted to 1. Feature plots of representative differential expressed genes were used to define the cell type.

The progenitors before splicing factor inhibitor treatment and the cells related with them after splicing factor treatment, which were traced by the CellTags and mitochondria mutations respectively, were merged into a eurat object. After transferring to the CellDataSet, choosing genes to define progress, reducing the dimensionality of the data and ordering the cells in pseudo-time were done respectively. The plot was colored by the pseudo-time scores and the cell type.

**Figure legends:**

**Figure S1.** Expression levels of splicing factors increased along with HSC expansion. **A** Representative FACS plot(left) and pie diagram(right) showed the percentage of HSPC subpopulations of human UCB HSCs after 7-day-cultured in vitro. Cell surface markers used to gate cell populations were listed. **B** Folds change of cell quantity compared with that of initial HSCs after 7-day-cultured in vitro. **C** Heatmap of splicing factors expression levels in HSCs, Multipotent Progenitors and Committed Progenitors. Many splicing factors gradually increased along with HSC differentiation. **D** Percentage variation of HSPC among samples treated with different PlaB concentrations, supplement to figure 1A. Data represented as means ± SDs from N = 3 duplicates. 2-tailed unpaired t test with unequal variance; n.s.: p > 0.05, *p < 0.05, **p < 0.01, ***p < 0.001.

**Figure S2.** Basic information of sc-RNAseq. **A, B** Distribution of confidently mapped reads information on scRNA-seq. scRNA-seq of the cells at both timepoints before and after PlaB treatment included 11,048 and 12,173 individual cells together with 2,933 and 3,054 median genes and 37,771 and 29,461 mean confidently mapped reads per cell. **C.** UMAP visualization based on 10× scRNA-seq before and after PlaB treatment. MD cells: Monocytes and Dendritic cells, EBM cells: Eosinophils, Basophils and Mast cells.

**Figure S3.** Basic information and lineage tracing by CellTagging. **A** The CellTagging workflow: a lentiviral construct contains an heritable 8-bp random CellTag barcode in the 3’ UTR of GFP, followed by an SV40 polyadenylation signal. Transduced cells express unique CellTags, enabling tracking of clonally related cells. **B** Number of CellTags detected in scRNA-seq samples. The number of celltag inserted in each cell was from 1 to 6 and the average number was 1. **C** Number of paired and individual CellTags detected in scRNA-seq samples before and after PlaB treatment. 7,888 cells before treatment and 9,570 cells after that have been detected with celltags and 6,290 cells between two timepoints were inserted with the same celltag. **D** Topological map of cells tracing by CellTagging, related with Figure 2D. Specific genes of each cluster were shown in PAGA layout.

**Figure S4.** Basic information of mitochondrial DNA mutation and lineage tracing. **A** The principle of lineage tracing by mitochondrial DNA mutation. Each cell has multiple mitochondria, which in turn contain many copies of mtDNA that may acquire somatic mutations over time. Correlation analysis of mutation patterns enable tracking of clonally related cells. **B** Based on the scRNA-seq data, distribution of coverage of the mitochondrial genome, numbers of mutations, frequency of mutations and numbers of mutations per cell were calculated. **C** Hierarchical clustering of mitochondrial genotyping profiles (rows) for cells on two timepoints. **D** Topological map of cells tracing by mitochondrial mutation, related with Figure 2D. Specific genes of each cluster were shown in PAGA layout.


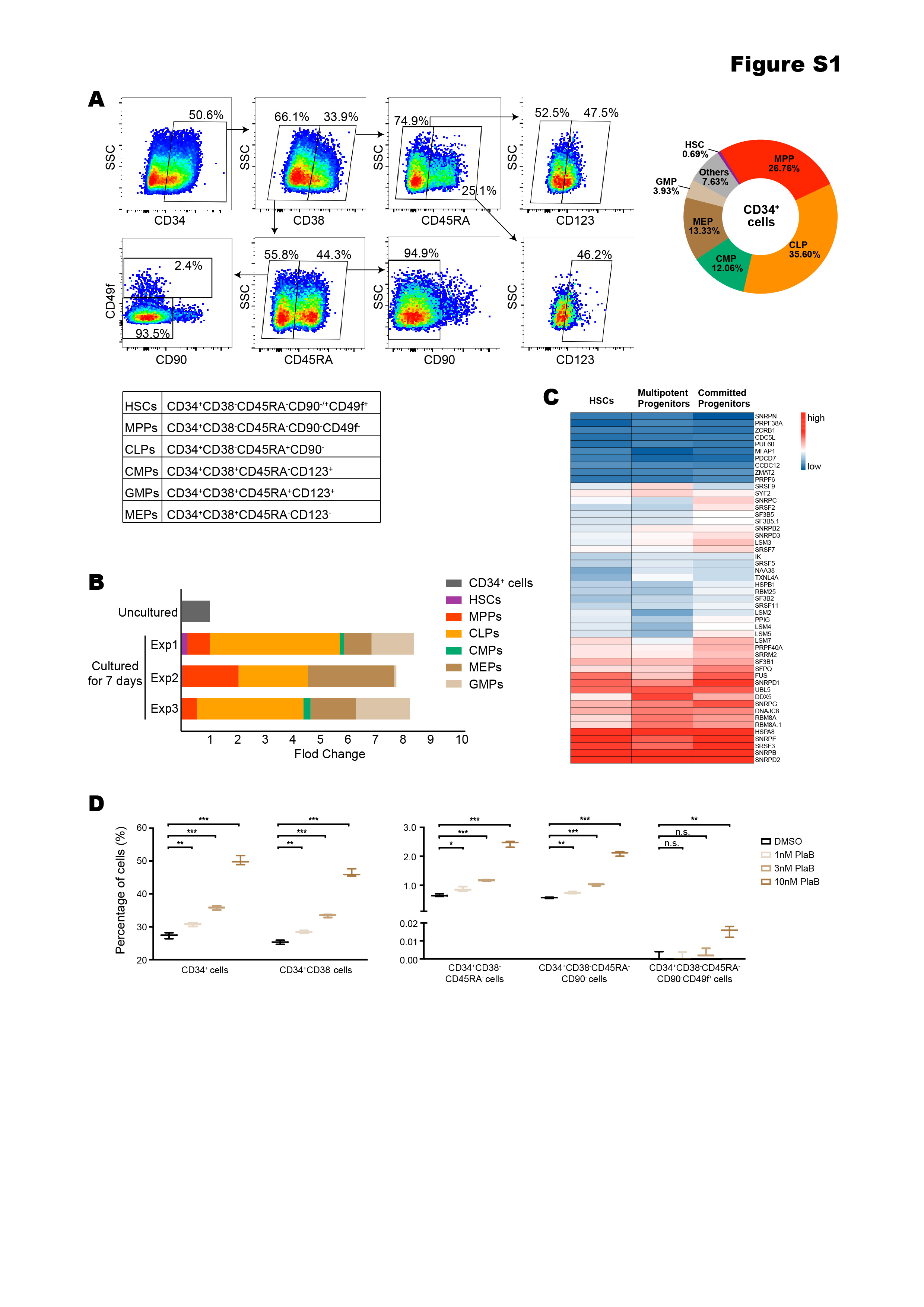

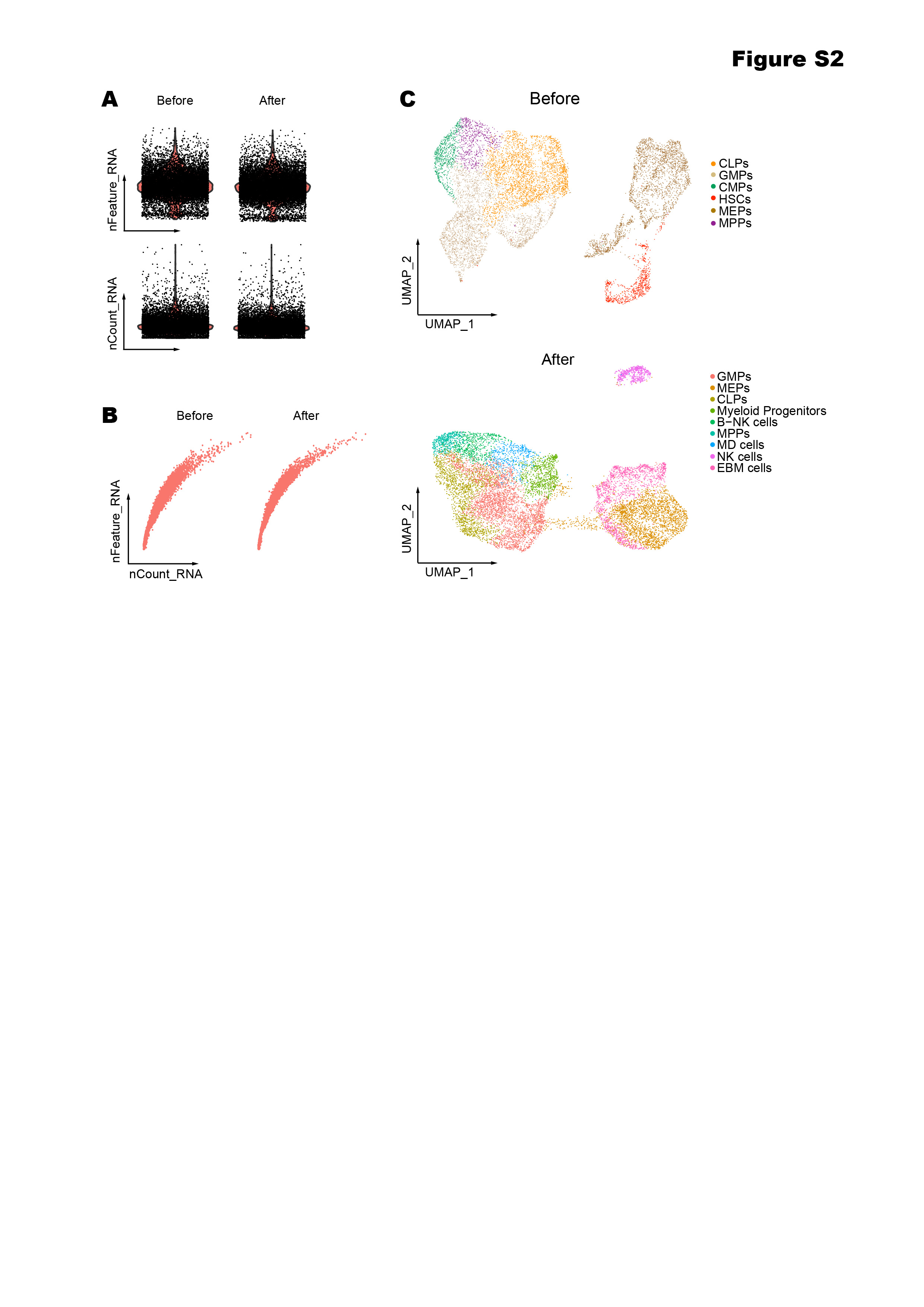

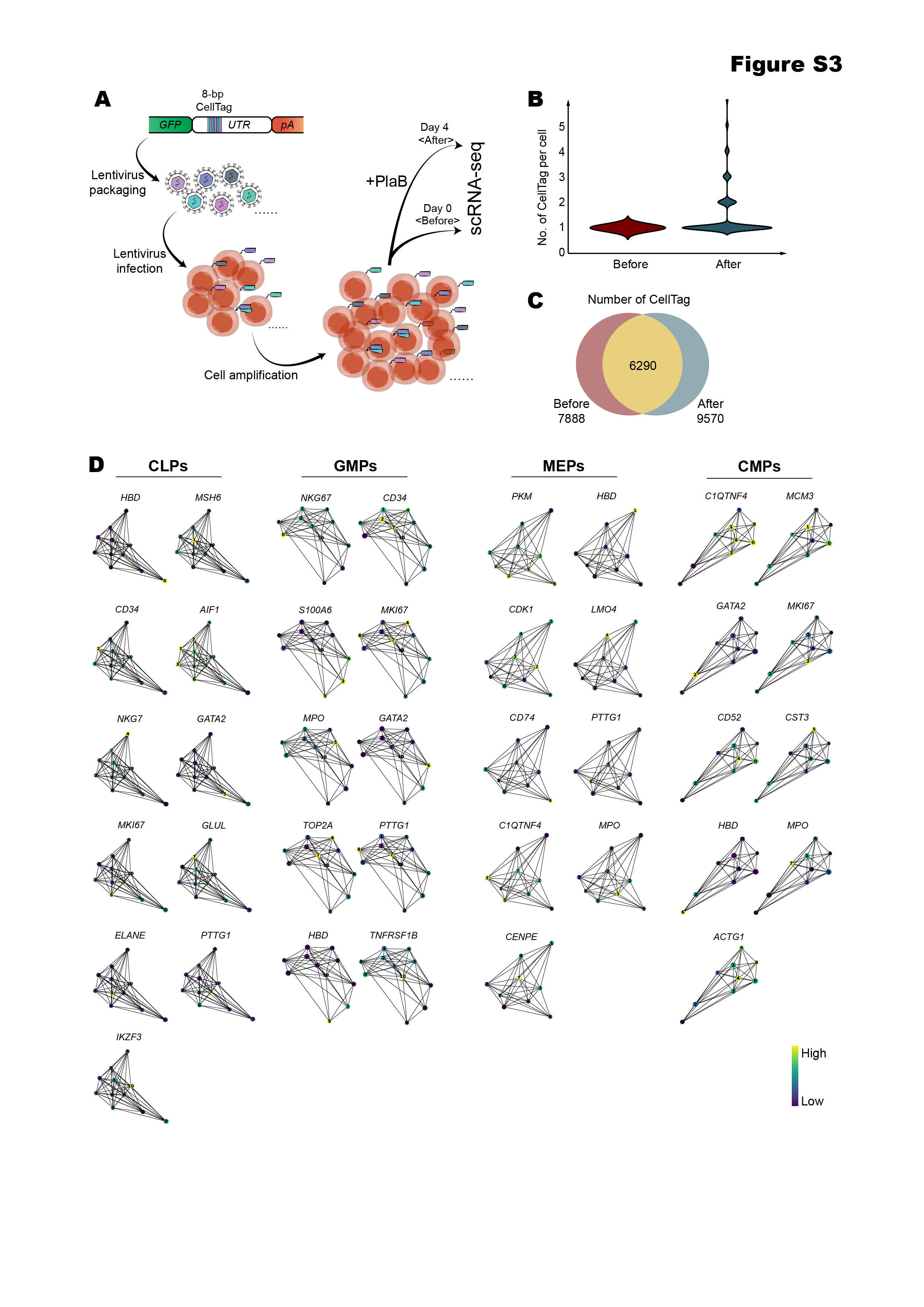

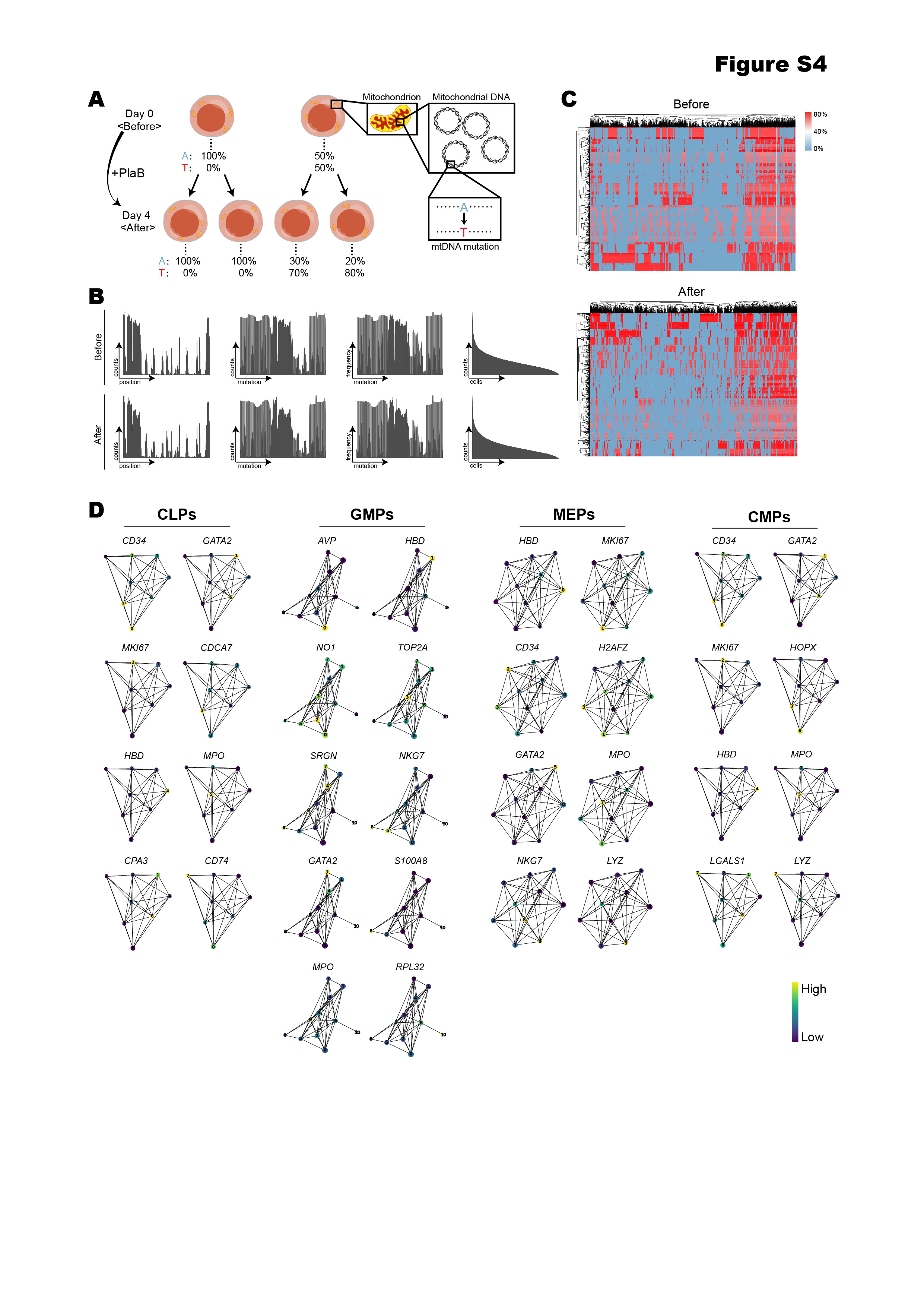

Supplement: Supplementary file 1 — Additional file 1: Figure S1. Expression levels of splicing factors increased along with HSC expansion. A Representative FACS plot(left) and pie diagram(right) showed the percentage of HSPC subpopulations of human UCB HSCs after 7-day-cultured in vitro. Cell surface markers used to gate cell populations were listed. B Folds change of cell quantity compared with that of initial HSCs after 7-day-cultured in vitro. C Heatmap of splicing factors expression levels in HSCs, Multipotent Progenitors and Committed Progenitors. Many splicing factors gradually increased along with HSC differentiation. D Percentage variation of HSPC among samples treated with different PlaB concentrations, supplement to figure 1A. Data represented as means ± SDs from N = 3 duplicates. 2-tailed unpaired t test with unequal variance; n.s.: p > 0.05, *p < 0.05, **p < 0.01, ***p < 0.001. Figure S2. Basic information of sc-RNAseq. A, B Distribution of confidently mapped reads information on scRNA-seq. scRNA-seq of the cells at both timepoints before and after PlaB treatment included 11,048 and 12,173 individual cells together with 2933 and 3054 median genes and 37,771 and 29,461 mean confidently mapped reads per cell. C UMAP visualization based on 10× scRNA-seq before and after PlaB treatment. MD cells: Monocytes and Dendritic cells, EBM cells: Eosinophils, Basophils and Mast cells. Figure S3. Basic information of CellTagging and lineage tracing. A The CellTagging workflow: a lentiviral construct contains a heritable 8-bp random CellTag barcode in the 3’ UTR of GFP, followed by an SV40 polyadenylation signal. Transduced cells express unique CellTags, enabling tracking of clonally related cells. B Number of CellTags detected in scRNA-seq samples. The number of celltag inserted in each cell was from 1 to 6 and the average number was 1. C Number of paired and individual CellTags detected in scRNA-seq samples before and after PlaB treatment. 7888 cells before treatment and 9570 cells after that have bee [file 40164_2022_288_MOESM1_ESM.docx]
